# Facial mask disturbs ocular exploration but not pupil reactivity

**DOI:** 10.3389/fnins.2022.1033243

**Published:** 2022-11-21

**Authors:** Vivien Rabadan, Camille Ricou, Marianne Latinus, Nadia Aguillon-Hernandez, Claire Wardak

**Affiliations:** UMR 1253, iBrain, Université de Tours, Inserm, Tours, France

**Keywords:** eye tracking (ET), pupil, face, emotion, mask, accessory, occlusion

## Abstract

**Introduction:**

The COVID-19 pandemic has imposed to wear a face mask that may have negative consequences for social interactions despite its health benefits. A lot of recent studies focused on emotion recognition of masked faces, as the mouth is, with the eyes, essential to convey emotional content. However, none have studied neurobehavioral and neurophysiological markers of masked faces perception, such as ocular exploration and pupil reactivity. The purpose of this eye tracking study was to quantify how wearing a facial accessory, and in particular a face mask, affected the ocular and pupillary response to a face, emotional or not.

**Methods:**

We used videos of actors wearing a facial accessory to characterize the visual exploration and pupillary response in several occlusion (no accessory, sunglasses, scarf, and mask) and emotional conditions (neutral, happy, and sad) in a population of 44 adults.

**Results:**

We showed that ocular exploration differed for face covered with an accessory, and in particular a mask, compared to the classical visual scanning pattern of a non-covered face. The covered areas of the face were less explored. Pupil reactivity seemed only slightly affected by the mask, while its sensitivity to emotions was observed even in the presence of a facial accessory.

**Discussion:**

These results suggest a mixed impact of the mask on attentional capture and physiological adjustment, which does not seem to be reconcilable with its strong effect on behavioral emotional recognition previously described.

## Introduction

Humans, from an early age, show a visual preference for the face (Turati et al., 2005). It is the most informative visual stimulus for social perception, allowing to determine the identity, the gender, the age as well as the emotional state of a person (Bruce and Young, 1986). Facial emotion recognition is an essential skill for living in a social world (Frith, 2009). Indeed, the ability to understand the emotions of others is crucial for good interpersonal relationships. Moreover, an incorrect emotion or identity recognition can interfere with normal social functioning and increase social anxiety (Davis et al., 2011).

Adults can be considered experts in facial processing (Carey, 1992). When a neurotypical person spontaneously observes a face, gaze travels over the eyes, mouth, and nose, thus forming a triangular exploration pattern (Vatikiotis-Bateson et al., 1998), with slight differences depending on gender (Coutrot et al., 2016), cultural context (Blais et al., 2008; Miellet et al., 2013), or individual recognition performance and cognitive abilities (Hsiao et al., 2022). These facial features, the eyes, the nose, and the mouth, have been shown to convey crucial information for face recognition (Keil, 2009), but also emotion recognition (Bassili, 1979), and are explored differently as a function of the emotional content of the face (Hernandez et al., 2009). To evaluate the importance of different facial areas on emotion recognition, studies have either displayed only face parts, blurred or filtered facial features, or displayed parts sequentially (Blais et al., 2012; Bombari et al., 2013; Meaux and Vuilleumier, 2016; Wegrzyn et al., 2017). If the eyes are crucial, these studies also revealed the importance of the mouth in emotion recognition (Blais et al., 2012), in particular for sadness (Bombari et al., 2013), or happiness (Wegrzyn et al., 2017). Other studies have focused on the ocular exploration of emotional faces, combined or not with a recognition task, and have shown that overall fixation time on the eye region is larger for fearful, angry and surprised faces while the mouth is more looked at for happy faces (Hernandez et al., 2009; Guo, 2012; Schurgin et al., 2014). Interestingly, a study combining eye-tracking with an emotional or identity comparison task showed that the lower part of the face is more explored when making an emotional judgment while the reverse was true for identity judgment (Malcolm et al., 2008).

Facial features are essential for face perception; however, face processing is not an analytic process based on isolated features. Indeed many studies have converged in showing that expert facial processing is holistic (Maurer et al., 2002), with the first-order (eyes above nose, and nose above mouth) and second-order (distance between features) relationships between facial features making the face an indivisible and coherent whole. This holistic facial processing therefore requires access to the entire face and raises the question about the effects of partial occlusion on facial exploration or emotion recognition. Many studies conclude that facial expression recognition is hindered when parts of the face are covered (Bassili, 1979; Roberson et al., 2012). Indeed, whether partial occlusion is due to glasses (Roberson et al., 2012) or a scarf (Kret and de Gelder, 2012), it represents an obstacle to reading different facial expression. Studies comparing the occlusion of the eyes and mouth regions showed that the identification of happy expressions is more disturbed by the occlusion of the mouth than the eyes, while for other emotions the results are not so clear. Kotsia et al. (2008) found anger was more disrupted by mouth occlusion and disgust by eye occlusion. Schurgin et al. (2014) reported the opposite trend. The addition of accessories or the occlusion with sunglasses, also has a negative impact on the recognition of unfamiliar faces (Graham and Ritchie, 2019). Nevertheless, accuracy is well above chance level, suggesting that the occlusion of an area does not abolish facial recognition capabilities.

As a result of the COVID-19 health crisis, a large part of the world population has been wearing a facial mask, and concerns about a negative impact of wearing a mask on social interactions have emerged (Saunders et al., 2021). Masks can easily disrupt our ability to reliably recognize or express emotions and information necessary for good communication during our daily social interactions (Marler and Ditton, 2021). Moreover, the surgical mask can have a negative psychological impact and induce stress and gloom in observers (Saint and Moscovitch, 2021; Saunders et al., 2021). Many studies on the effect of observing a masked face have recently been carried out. As in previous occlusion studies, facial expression recognition seems affected. Noyes et al. (2021) contrasted faces wearing a mask or sunglasses with bare faces on several emotions recognition and showed a decrease in emotion recognition accuracy when the mouth was masked. Carbon (2020) and Freud et al. (2020) showed that emotional identification was strongly disturbed by the presence of a mask, in particular for sadness. However, many of these studies used digitally added masks to existing emotional face photos, lacking naturalness. Moreover, few studies on masked face perception used dynamic stimuli (videos), although this realistic aspect plays a key role in the discrimination of different emotions (Blais et al., 2012). Dynamism is indeed considered as an important component of naturalistic stimuli (Richoz et al., 2018) and impact physiological arousal (Aguillon-Hernandez et al., 2020). To take this aspect into account, we created a set of videos of actors displaying different emotions (neutral, happiness, and sadness), filmed either bare face or while really wearing several facial accessories (sunglasses, scarf, and surgical mask). With these new controlled ecological videos, we demonstrated that real-worn masks impacted emotion recognition (Aguillon-Hernandez et al., 2022). We found an effect of mask on visual emotion recognition with a loss of accuracy of 17% for happiness and 25% for sadness. Importantly, we had no effect of sunglasses and an effect of scarf only on sadness recognition (Aguillon-Hernandez et al., 2022).

While occlusion has a clear effect on emotion recognition, it is not clear whether this is related to the reduced amount of available information (thus thwarting ocular exploration), a reduced attentional capture, a disturbed holistic processing, or an altered physiological arousal. Several of these processes can be inferred from eye-tracking studies. Indeed, ocular exploration of a scene is guided both by low-level, bottom-up information (for example, movement, color), and several top-down factors like expectation, internal representations, and social information for example (Flechsenhar and Gamer, 2017). This information would be combined in a saliency map or priority map guiding attention and eye movements (e.g., Treue, 2003). To our knowledge, only one study looked at the modulation of visual exploration patterns by surgical masks (Hsiao et al., 2022) and reported eye-focused exploration patterns in masked faces. These results need to be extended to emotional and dynamic faces, and contrasted with other facial accessories.

Recorded simultaneously with ocular exploration, pupil diameter variation is another interesting marker of facial processing (Martineau et al., 2011; Aguillon-Hernandez et al., 2020). Pupillary dilation can be used as a physiological marker of social or affective arousal in response to the presentation of faces, emotional or not (Ekman, 1992). Indeed, evoked pupil responses are strongly correlated with the activity of the noradrenergic nuclei of the locus coeruleus (Joshi et al., 2016), linked to the attentional engagement or arousal of a subject (Sara and Bouret, 2012). However, pupil dilation exhibit slow dynamics and cannot easily distinguish successive processing or cognitive steps, thus integrating many inputs like sensory saliency, cognitive representations or emotion processing (e.g., Joshi and Gold, 2020). Previous work has shown that faces with emotional valence yield greater pupil dilation (Bradley et al., 2008), exacerbated for negative valence emotions (Yrttiaho et al., 2017; Aguillon-Hernandez et al., 2020). A study by Aguillon-Hernandez et al. (2020) highlighted physiological adjustment to ecological social stimuli, with larger pupil dilation for social (neutral and emotional faces) compared to non-social stimuli and for dynamic stimuli (videos of faces) compared to static stimuli (photos of faces).

The goal of this study was to quantify how wearing a facial accessory, and in particular a COVID-19 mask, affected ocular and pupillary responses to the observation of a face, emotional or not. We used videos previously created and behaviorally evaluated (Aguillon-Hernandez et al., 2022), featuring four facial conditions (no accessory, sunglasses, tube scarf, and COVID-19 mask) and three emotional conditions (neutrality, happiness, and sadness). The comparison of the mask and scarf conditions aimed to dissociate the effect of the occlusion of the lower part of the face from a possible negative psychological impact specific to the surgical mask (Saunders et al., 2021). In order to measure spontaneous responses, as close as possible from a real ecological interaction, we did not ask any judgment about emotion recognition. As the accessories masked the main regions of interest of the face (eyes or mouth), we expected ocular exploration to be altered in the presence of an accessory, maybe redirecting gaze toward the visible part of the face (as observed by Hsiao et al., 2022). For the pupillary response, we expected a greater dilation for emotional faces compared to neutral faces as already described (Aguillon-Hernandez et al., 2020). This response could be reduced in the presence of an accessory, in particular masking the mouth, as emotions are less recognized in this condition (Aguillon-Hernandez et al., 2022).

## Materials and methods

### Participants

We recruited 44 participants (22 females), aged 18–35 (mean = 23.23 ± std 3.26 year). This age range was chosen to minimize variations in basal pupil diameter (Fairhall et al., 2006). Exclusion criteria were abnormal or uncorrected vision, personal history of psychiatric or neurological disorders, and personal history of learning difficulties, difficulties in learning to walk or speak. Written informed consent was obtained prior to the experiment. The study was approved by an Ethics Committee (CPP, protocol PROSCEA 2017-A00756-47) and conformed with the Declaration of Helsinki (World Medical Association, 2013).

### Stimuli and protocol

**FIGURE 1:**
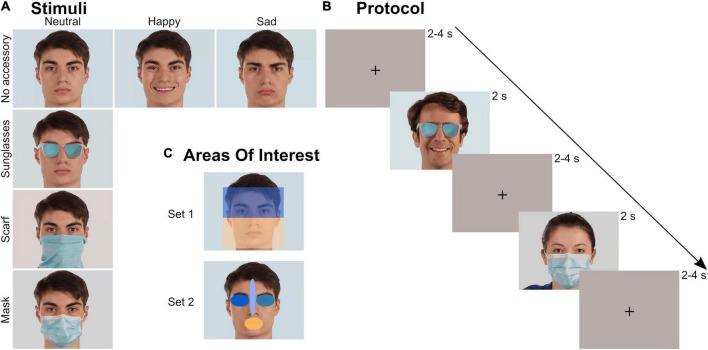
Stimuli and protocol. **(A)** Stimuli: videos of four actors (2 males) with four occlusion and three emotion conditions were tested. **(B)** Protocol: each video lasted 2 s and was preceded by a 2–4 s inter-trial uniform image with a black cross. The 48 videos were randomly presented during a block. Three blocks were recorded by participant. **(C)** Areas of Interest (AOI): two sets of AOIs were created to analyze the ocular exploration. Within each set, the different AOIs covered the same area.

The stimulus set was evaluated in a previous study (Aguillon-Hernandez et al., 2022) focusing on explicit behavioral emotion recognition. It was composed of videos of faces, expressing different realist emotions and wearing or not a facial accessory. The accessory covered either the upper part of the face (sunglasses) or the lower part of the face (mask and tube scarf, sometimes called neck gaiter). A total of 48 videos were tested: 4 actors (2 males) × 3 emotions (neutrality/happiness/sadness) × 4 facial occlusion conditions (no accessory/sunglasses/scarf/mask) (Figure 1A). Each video started with an actor in a neutral state that either stayed neutral or performed an emotion before returning to neutral. The emotional content of the videos without accessory was validated both objectively (FaceReader^®^, FR6, Noldus, Wageningen, The Netherlands) and subjectively (Aguillon-Hernandez et al., 2022). All videos were processed to last 2 s, to frame each face identically, and to be matched in colorimetry. Global luminosity of the stimuli and the room were controlled and kept constant (25 Lux) for each participant. The stimuli were presented in the center of a 27-inch monitor with a resolution of 1,920 × 1,080 px. In-between each video, an inter-trial image composed of a uniform background and a central black cross (located at the level of the upper nose, Figure 1B) was presented, matched in colorimetry and luminosity with the videos.

The set of 48 videos was presented three times to each participant. For each block, the order of presentation was randomized. Inter-trial stimulus interval was between 2 and 4 s (Figure 1B). No instructions were given to the participants except to look at the screen and remain silent.

### Data acquisition and processing

Data were acquired with a Tobii^®^ Pro Fusion eye tracker (Tobii, Stockholm, Sweden; sampling rate of 250 Hz), with an accuracy of 0.3° and a precision of 0.04° in optimal conditions. The protocol was run with Tobii^®^ Pro Lab. Each participant was installed in a comfortable armchair in front of the monitor at a distance of about 70 cm (distance calculated by the eye tracker: 57.3–78.2 cm). Before each block of videos, a nine-point calibration procedure was performed using animated circles to attract the gaze.

The ocular exploration of the videos was analyzed through several parameters depending on Areas of Interest (AOIs). We created several AOIs: first the whole screen, to check that all the videos were equally explored, then two different sets of AOIs (Figure 1C). Within each set, all AOIs had the same surface (rectangles in Set 1: 320,000 px; ellipses in Set 2: 30,278 px). Set 1 divided the face into two large parts: the upper part (containing the eyes), and the lower part (containing the whole surgical mask). Set 2 consisted of four elliptical AOIs located on the mouth, the right eye, the left eye, and the space between the eyebrows including the tip of the nose. We mainly analyzed the time spent (in s) within those AOIs, relatively to the total time spent on the screen, as computed by Tobii Pro Lab (every valid eye tracking sample). We also analyzed the latency of the first entry (in ms) in each AOI of Set 2.

For pupil analysis, we extracted raw data from Tobii and processed the signal using in-house MATLAB scripts. Some data loss was observed for some participants. Except in one subject, data loss always lasted less than the duration of a blink (200–300 ms) and was interpolated to its nearest values. For the subject with longer data loss, trials (*n* = 2) with lost data were removed. Blinks and signal artifacts were identified thanks to a velocity threshold and pupil diameter values were replaced by the median values of a pre- and post-blink 120 ms interval. Then, we applied a median filter to remove signal artifacts and smooth the signal. Residual blinks were visually identified and manually interpolated. For each trial (starting at video onset), a baseline pupil size was calculated by taking the median value of the pupil diameter recorded over the last 200 ms before the video onset. This baseline value was subtracted from the pupillary diameter recorded during the 4 s after the start of the video presentation (2 s of video and 2 s of inter-trial). For each participant, a mean time course was calculated for each of the 12 categories (3 emotions × 4 accessories). We extracted several parameters from these time courses: the dilation peak amplitude (maximum pupillary diameter between 1.1 and 3.4 s, in mm) and its latency (in ms).

### Statistical analyses

Statistical analyses were carried out using the software Statistica13^®^. For all parameters, normality of the distribution and homogeneity of variance were verified with the Kolmogorov–Smirnov and Levene tests.

The influence of the accessory (×4: no accessory, sunglasses, scarf, and mask) on the different parameters according to the emotion (×3: neutral, happy, and sad) and the AOIs for ocular exploration (×2 for Set 1 and ×4 for Set 2), was evaluated with a repeated measure ANOVA within the General Linear Model (GLM) Framework, corrected by Greenhouse–Geisser and completed by *post-hoc* corrected planned multiple comparisons. Pupil time courses were also analyzed with a GLM, adding the effect of time (×8 time points: one mean value for each 500 ms time interval), with Bonferroni multiple comparisons. *P*-corrected values and effect size (η^2^) are provided for each significant effect.

Without any *a priori* hypothesis about the statistical size effect expected, we performed *a posteriori* G*Power^®^ 3.1 sensitivity analysis. We evaluated we could expect a small effect size of *f* = 0.15 (η^2^ = 0.022) according to the size of our population (*n* = 44), an error probability of 0.05 and a power of 0.95.

## Results

### Ocular exploration of faces

**FIGURE 2:**
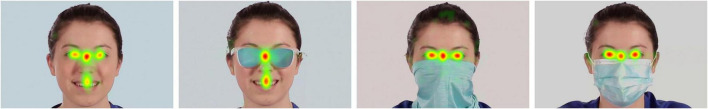
Example of heat maps for the exploration of a happy face. The mean time spent for all participants is represented by a color gradient from green (low time spent) to red (high time spent).

We analyzed how the participants explored the videos of the faces, depending on the accessory worn (or not) and the emotion displayed. Qualitatively, we observed the classical ocular pattern when exploring a bare face (i.e., mainly exploration of the eyes and the mouth; see an example in Figure 2). This pattern was modified by the presence of an accessory. To quantify these observations, we analyzed the effect of three factors: accessory, emotion and AOI on the time spent within several AOIs.

**FIGURE 3:**
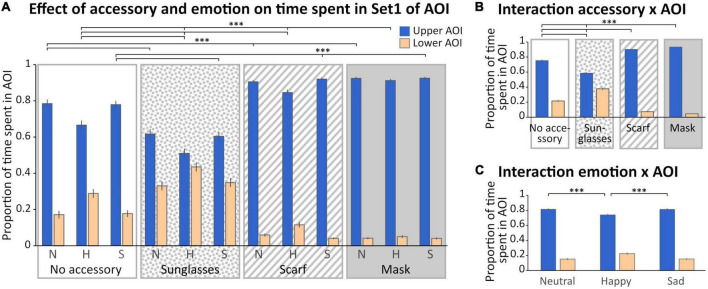
Analysis of the time spent in Set 1 of AOI. **(A)** Mean (± standard error) of the proportion of time spent (relative to the time spent on the screen) in the Areas of Interest (AOIs) (upper AOI: blue, left columns; lower AOI: yellow, right columns) according to accessory (no accessory: white background; sunglasses: dotted background; scarf: hatched background; mask: gray background) and emotion (neutral: N, happy: H, and sad: S). **(B)** Mean (± standard error) of the proportion of time spent in the AOIs illustrating the accessory × AOI interaction. **(C)** Mean (± standard error) of the proportion of time spent in the AOIs illustrating the emotion × AOI interaction. For sake of clarity, only the significant comparisons for the upper AOI are illustrated. The pattern is identical for the lower AOI. ^***^p < 0.001.

With AOIs of Set 1 (Figures 1C, 3), we observed a main effect of the accessory [*F*_(3_,_132)_ = 3.96; *p* < 0.01, η^2^ = 0.08], with a time spent in AOIs in the scarf and mask conditions significantly lower than in the sunglasses condition (*p* < 0.05 for both), and a main effect of the AOI [*F*_(1_,_44)_ = 268.16; *p* < 0.0001, η^2^ = 0.86], with a greater time spent in the upper AOI. Three interactions were also significant (“Accessory × AOI,” “Emotion × AOI,” and “Accessory × Emotion × AOI”).

First, we obtained a significant “Accessory × AOI” interaction [*F*_(3_,_132)_ = 100.47; *p* < 0.001, η^2^ = 0.69; Figure 3B]. Masking the lower part of the face, with a mask or a scarf, significantly increased the time spent in the upper AOI compared to the sunglasses and no accessory conditions (*p* < 0.001 for each comparison) and decreased the time spent in the lower AOI (scarf < sunglasses and no accessory, mask < sunglasses and no accessory, *p* < 0.001 for each comparison). Finally, the sunglasses biased the exploration toward the lower part of the face compared to the no accessory condition (*p* < 0.001 for each comparison).

Secondly, we obtained a significant “Emotion × AOI” interaction [*F*_(2_,_88)_ = 81.684; *p* < 0.001, η^2^ = 0.65; Figure 3C]: in the happy condition the exploration was biased toward the lower part of the face compared to neutrality and sadness (*p* < 0.001 for each comparison and each AOI).

Finally, we observed a significant “Accessory × Emotion × AOI” interaction [*F*_(6_,_264)_ = 10.171; *p* < 0.001, η^2^ = 0.19; Figure 3A]. In the happy condition, the time spent in the lower AOI was higher when wearing sunglasses and decreased depending on the accessory (sunglasses > no accessory > scarf > mask, *p* < 0.001 for all comparisons); the reverse pattern was observed for the upper AOI (sunglasses < no accessory < scarf < mask, *p* < 0.001 for all comparisons). For both the sad and neutral conditions (which did not differ), the time spent in the lower AOI was higher when wearing sunglasses than no accessory, and in the no accessory condition compared to both the scarf and mask conditions (which did not differ; sunglasses > no accessory > scarf = mask, *p* < 0.001 for the significant comparisons); the reverse pattern was observed for the upper AOI (sunglasses < no accessory < scarf = mask, *p* < 0.001 for all significant comparisons).

**FIGURE 4:**
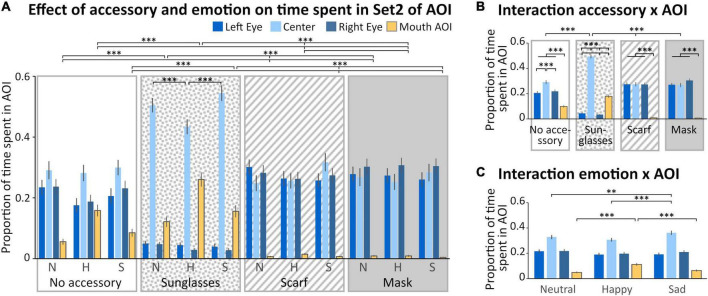
Analysis of the time spent in Set 2 of AOI. **(A)** Mean (± standard error) of the proportion of time spent (relative to the time spent on the screen) in the Areas of Interest (AOIs) (left eye: medium blue, first columns; center: light blue, second columns; right eye: dark blue, third columns; mouth: yellow, last columns) according to accessory (no accessory: white background; sunglasses: dotted background; scarf: hatched background; mask: gray background) and emotion (neutral: N, happy: H, and sad: S). For sake of clarity, only the significant comparisons for the mouth AOI are illustrated. The pattern is identical for the left and right eyes AOIs, except for the scarf vs mask comparison (see Results section). We also illustrated the comparisons within the sunglasses condition. **(B)** Mean (± standard error) of the proportion of time spent in the AOIs illustrating the accessory × AOI interaction. **(C)** Mean (± standard error) of the proportion of time spent in the AOIs illustrating the emotion × AOI interaction. ^**^p < 0.01; ^***^p < 0.001.

In the Set 2 of AOIs (Figure 1C), four regions were analyzed (left and right eyes, mouth and space between the eyes, Figure 4). We observed a main effect of the accessory [*F*_(3_,_132)_ = 11.943; *p* < 0.001, η^2^ = 0.21], with a time spent in AOIs significantly lower when wearing sunglasses compared to the other accessory conditions (*p* < 0.001 for all comparisons). We also observed a main effect of the AOI [*F*_(1_,_132)_ = 25.224; *p* < 0.001, η^2^ = 0.36], with a higher time spent within the center AOI compared to the three other AOIs (*p* < 0.001 for all comparisons), and a lower time spent in the mouth AOI compared to the three other AOIs (*p* < 0.001 for all comparisons). No significant difference was observed between the time spent on the left or right eye. Four interactions (“Accessory × Emotion,” “Accessory × AOI,” “Emotion × AOI,” and “Accessory × Emotion × AOI”) were significant.

First, we obtained a significant “Accessory × Emotion” interaction [*F*_(6_,_264)_ = 3.8022; *p* < 0.01, η^2^ = 0.08]. In the sad and neutral conditions, the time spent in AOIs was lower for the sunglasses than for the three other accessory conditions (*p* < 0.01). When wearing a scarf, the time spent in AOIs was significantly lower in the happy compared to sad condition (*p* < 0.01). When wearing sunglasses, the time spent in AOIs was significantly lower in the neutral compared to happy condition (*p* < 0.01).

Secondly, we obtained a significant “Accessory × AOI” interaction [*F*_(9_,_396)_ = 80.557; *p* = 0.002, η^2^ = 0.65; Figure 4B]. For the mask, scarf and no accessory conditions, the time spent in the mouth AOI was lower than for the three other AOIs (*p* < 0.001 for all comparisons). For the no accessory condition, the time spent in the center AOI was higher than for the three other AOIs (*p* < 0.001 for all comparisons). For the sunglasses condition, the time spent in the center AOI was higher than in the mouth AOI, which was higher than in the two eyes AOIs (center > mouth > left and right eyes, *p* < 0.001 for all comparisons). The center AOI was more fixated in the sunglasses condition than in the other accessory conditions (*p* < 0.001 for all comparisons).

Thirdly, we obtained a significant “Emotion × AOI” interaction [*F*_(6_,_264)_ = 24.690; *p* < 0.001, η^2^ = 0.36; Figure 4C]. Specifically, we observed that the time spent in the center AOI in the sad condition was higher than for happy (*p* < 0.001) or neutral (*p* < 0.01) conditions. Moreover, the time spent in the mouth AOI was higher in the happy than in the sad and neutral conditions (*p* < 0.001 for both comparisons).

Finally, we obtained a significant “Accessory × Emotion × AOI” interaction [*F*_(18_,_792)_ = 9.5766; *p* < 0.001, η^2^ = 0.18; Figure 4A]. In the happy condition, the time spent in the mouth AOI was higher when wearing sunglasses and decreased depending on the accessory (sunglasses > no accessory > scarf > mask, *p* < 0.001 for all comparisons). The reverse pattern was observed for the left and right eye AOIs, except that there was no difference between the scarf and the mask conditions (sunglasses < no accessory < scarf = mask, *p* < 0.001 for the significant comparisons). For both the sad and neutral conditions (which did not differ), the time spent in the mouth AOI was higher when wearing sunglasses than other accessories, and in the no accessory condition compared to both the scarf and mask conditions (which did not differ; sunglasses > no accessory > scarf = mask, *p* < 0.001 for the significant comparisons); the reverse pattern was observed for the left and right eye AOIs (sunglasses < no accessory < scarf = mask, *p* < 0.001 for all significant comparisons). Moreover, when wearing sunglasses, the time spent in the center AOI was lower in the happy compared to sad and neutral conditions (*p* < 0.001 for both comparisons).

To go further in the analysis of the exploration pattern, we also analyzed the latency of the first entry within the four AOIs of Set 2. We hypothesized that the time spent in the center AOI may reflect the fact that the fixation cross present during inter-trial was located within this AOI and, as a consequence, the exploration always started from that location. Indeed, participants’ gaze was almost always located within the center AOI at the beginning of the exploration, as the mean latency of the first entry in this AOI was 54 ms. We analyzed the effect of accessory and emotion on the latency of the first entry within the three other AOIs. We observed a main effect of the accessory [*F*_(3_,_27)_ = 12.44; *p* < 0.001, η^2^ = 0.58], with a latency of the first entry significantly longer when wearing sunglasses compared to the other accessory conditions (*p* < 0.001 for all comparisons; mean latency of the first entry ± Std: sunglasses 1,006 ± 465 ms, no accessory 880 ± 420 ms, mask 833 ± 469 ms, scarf 829 ± 437 ms), i.e., a longer fixation within the center AOI before exploring the face. We also observed a main effect of the AOI [*F*_(2_,_18_ = 11.56; *p* < 0.01, η^2^ = 0.56], with the left eye AOI being explored first (*p* < 0.001 compared to right eye AOI, *p* < 0.05 compared to the mouth AOI; left eye AOI 785 ± 432 ms, right eye AOI 895 ± 448 ms, mouth AOI 982 ± 458 ms). We obtained a significant “Accessory × AOI” interaction [*F*_(6_,_54)_ = 12.62; *p* < 0.0001, η^2^ = 0.58], reflecting the fact that masking the lower part of the face (scarf and mask conditions, that did not differ) delayed the exploration of the mouth AOI (*p* < 0.05 for all comparisons; no significant difference between the sunglasses and no accessory conditions) and masking the upper part of the face (sunglasses) delayed the exploration of the eyes (*p* < 0.05 for all comparisons; no significant difference between the scarf, mask and no accessory conditions). However, we found no significant effect of emotion, nor any significant interaction involving emotion. To summarize, accessories not only affected the cumulated time spent in the different AOIs, but also affected the spatial strategy of exploration, with the non-masked regions explored first.

### Pupillary reactivity to faces

**FIGURE 5:**
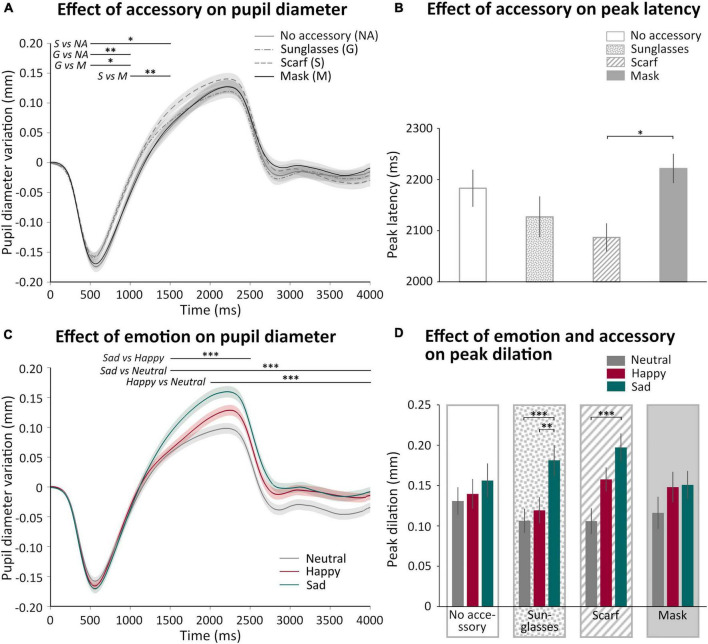
Effect of accessory and emotion on pupil variations. **(A)** Mean (± standard error) pupil variation (mm) of all participant during 4 s according to accessory (no accessory NA: solid gray, sunglasses G: dash point gray, scarf S: dashed gray, and mask M: solid black). **(B)** Mean (± standard error) peak latency (ms) of all participant according to accessory (no accessory: white; sunglasses: dotted; scarf: hatched; mask: gray). **(C)** Mean (± standard error) pupil variation (mm) of all participant during 4 s according to emotion (neutral: gray; happy: burgundy; sad: green). **(D)** Mean (± standard error) peak dilation (mm) of all participant according to accessory (no accessory: white, first panel; sunglasses: dotted, second panel; scarf: hatched, third panel; mask: gray, last panel) and emotion (neutral: gray, left columns; happy: burgundy, middle columns; sad: green, right columns). *p < 0.05; ^**^p < 0.01; ^***^p < 0.001.

We analyzed how the pupil diameter varied as a function of the accessory and the emotion displayed in the videos. Figure 5 shows the mean pupil variation as a function of the accessory (Figure 5A) or of the emotion (Figure 5C) during 4 s (the first 2 s corresponded to the video presentation, followed by 2 s of inter-trial stimulus). We observed a rapid pupil constriction followed by a pupil dilation and, after the end of the video, a return to baseline. The analysis of the time courses showed that we indeed obtained a main effect of time [*F*_(7_,_301)_ = 106.08, *p* < 0.0001, η^2^ = 0.71], with the first 500 ms significantly different from all the other 500 ms windows until 3,500 ms (*p* < 0.0001 for all comparisons, except 0–500 ms vs. 3,000–3,500 ms *p* < 0.05). First, we observed a significant “Accessory × Time” interaction [*F*_(21_,_903)_ = 4.40, *p* < 0.001, η^2^ = 0.09], illustrating early differences in the time courses (Figure 5A), with the sunglasses and scarf conditions eliciting earlier dilation than the two other conditions (scarf > no accessory *p* < 0.05 500–1,500 ms, scarf > mask *p* < 0.01 1,000–1,500 ms, sunglasse > no accessory *p* < 0.01 500–1,000 ms, sunglasses > mask *p* < 0.05 500–1,000 ms). The analysis of the time courses also revealed a main effect of emotion [*F*_(2_,_86)_ = 7.42, *p* < 0.01, η^2^ = 0.15], with the sad condition significantly different from the neutral condition (*p* < 0.001). A significant “Emotion × Time” interaction [*F*_(14_,_602)_ = 13.51, *p* < 0.0001, η^2^ = 0.24] revealed that the sad condition produced a larger dilation than the happy condition, itself larger than the neutral condition, in different time windows (Figure 5C; sad > happy *p* < 0.0001 for 1,500–2,500 ms; sad > neutral *p* < 0.0001 for 1,500–4,000 ms; happy > neutral *p* < 0.0001 for 2,000–4,000 ms).

We were interested in analyzing pupil dilation, *a priori* reflecting the cognitive and emotional content of the video. We thus extracted the amplitude (peak dilation) and latency (peak latency) of the peak pupil dilation. We analyzed the effect of the accessory and the emotion on peak dilation. There was no main effect of the accessory, but a main effect of the emotion [*F*_(2_,_86)_ = 21.71; *p* < 0.001, η^2^ = 0.33]. Indeed, as observed on the time courses (Figure 5C), peak dilation was significantly higher for sadness compared to happiness (*p* < 0.01) and neutrality (*p* < 0.001). We also observed a significant “Accessory × Emotion” interaction [*F*_(6_,_258)_ = 2.48; *p* < 0.024, η^2^ = 0.05]. For both the scarf and sunglasses conditions, peak dilation was larger in the sad compared to the neutral condition (*p* < 0.001 for both comparisons), while the comparison with the happy condition was significant only when wearing sunglasses (*p* < 0.01). However, there was no significant difference in the peak dilation induced by sadness for the different accessory conditions. We also observed a small significant effect of the accessory on peak latency [*F*_(3_,_129)_ = 3.048; *p* < 0.031, η^2^ = 0.07; Figure 5B], with a longer latency for the mask compared to the scarf conditions (*p* < 0.05), while there was no effect of emotion nor an “Accessory × Emotion” interaction on pupil peak latency. We tested the correlation between peak latency and peak dilation, these two parameters were not correlated.

## Discussion

In this study, we examined the influence of facial accessories, and in particular the face mask, on the ocular behavior and pupillary reactivity in response to emotional and non-emotional faces. We observed a significant impact of both the accessories and the emotional content on the ocular exploration of the face, but mainly an effect of emotion on pupil dilation.

The ocular exploration of a face wearing an accessory was modified compared to the classical exploration pattern (Vatikiotis-Bateson et al., 1998; Blais et al., 2008; Miellet et al., 2013; Coutrot et al., 2016) found in the no accessory condition. When we considered the time spent on the whole face (Set 1 of AOIs), the upper part of the face was always the most visited but the time spent in this AOI was influenced by the accessory. As expected, covering the lower part of the face (by a scarf or a mask) decreased the time spent on the lower part and increased the time spent on the upper part of the face; conversely, covering the upper part of the face (with sunglasses) increased the time spent in the lower part of the face. Both are consistent with Hsiao et al. (2022) who suggested that ocular movements during a face recognition task were guided by the visual information available, mainly the eyes region when the face is masked. Reduced low-level visual input would indeed decrease the saliency of the masked parts of the face, thus capturing less attention and gaze. A more precise spatial analysis (Set 2 of AOIs) revealed interesting exploration patterns in the upper and lower parts of the face. Indeed, the time spent on the upper part of the face was not focused only on the eyes: there was a bias toward the space between the eyes, more pronounced when observing a face wearing sunglasses and absent when the lower part of the face was covered. So, while the upper part of the face was still more explored when observing somebody wearing sunglasses, the eyes region was not explored. The exploration of the space between the two eyes was already described by Schurgin et al. (2014) but should be interpreted with caution. Indeed, this bias could be explained by the location of the fixation cross during the inter-trial: in our study as in Schurgin’s, before the presentation of the face, a cross was displayed allowing for the ocular exploration of the face always to start from the same location. As a result, all participants spent time at this location at least at the start of each trial, as confirmed by the analysis of the latency of the first entry in the center AOI. In the scarf and mask conditions, the inter-eyes region was less explored than in the no accessory condition, potentially reflecting a less dispersed exploration. The time spent on the lower part of the face was focused on the mouth when it was visible, but, as could be expected, was more dispersed when the mouth was covered. In the sunglasses condition, with no visible eyes to explore, the analysis of the latency of the first entry in the other AOIs suggested a longer disengagement from this location. We had hypothesized that, the eyes being considered as more salient than the mouth (Pesciarelli et al., 2016), when they are masked by sunglasses the gaze would be attracted toward the next most salient part of the face, i.e., the mouth. While indeed the mouth was overall more explored in this condition, the dynamics of the exploration (as indexed by the latency of the first entry in the AOI) revealed that the mouth AOI was not visited more quickly. The longer disengagement from the center location at the beginning of the exploration in the sunglasses condition could thus reveal either a low saliency of the unmasked parts of the face, or more probably a perturbation of the prototypical exploration strategy starting on the eyes. Guo (2007) has proposed that, in the context of face exploration, non-human primates always explore the eyes’ location first, even when the content of that region was modified. Our data do not allow to test completely this hypothesis as the exploration always starts from the location between the eyes, which could be considered as within the overall eye region. On the other hand, a recent model suggested that low-level saliency influenced only the first saccade, all subsequent saccades being better explained by top-down factors (Schütt et al., 2019). Our results suggest that the dynamic of this first saccade can still be influenced by internal factors.

To our knowledge, visual exploration of emotional faces wearing accessories has not yet been studied. Interaction between the accessories and the emotional content mainly reflected an effect of accessory on happy faces: the lower part of the face and the mouth were more explored when the faces were smiling (Guo, 2012; Schurgin et al., 2014), with a larger effect when the eyes were covered and a smaller effect when the mouth was covered. While we interpret these results as an impact of the emotional content of the stimulations, we cannot rule out that the time spent on the mouth in the happy condition may be explained at least partly by low-level local movement information attracting the gaze. This effect was not found on the latency of the first entry in the AOIs, possibly because of the dynamic nature of the stimuli (the smile was not visible at the beginning of the video). The only effect specific to the mask was that the time spent on the mouth in the happy condition was lower compared to that of the scarf condition, and did not differ from the other emotional conditions. The difference for the scarf and the mask in the happy condition could be linked to the specific decrease in happiness recognition in our previous behavioral study (Aguillon-Hernandez et al., 2022) for faces wearing a mask. It is possible that the tube scarf we used for the videos still allowed access to some movement information, preserving happiness recognition and the exploration bias toward the mouth (when compared to the other emotions). Note that the results differed between ocular exploration and behavioral responses using the same stimuli (Aguillon-Hernandez et al., 2022). Indeed, we observed a decreased performance for sadness recognition in the mask and scarf conditions, while the visual strategy did not seem to differ between the neutral and sad faces in the present study. The exploration strategies observed made the best of the available information on the face. It is not clear if the results would have been different if participants had explicitly been asked to judge the emotional content of the videos while the ocular exploration was recorded. The exploration pattern obtained for non-covered faces was similar to the pattern obtained in studies combining visual exploration measurement and emotional recognition task (Guo, 2012; Schurgin et al., 2014). Moreover, in his study, Guo reduced the intensity of emotions on the faces, inducing a decreased recognition performance, without exploration pattern modifications. Note however, that a slight change in ocular strategy cannot be excluded when given an explicit recognition instruction (Malcolm et al., 2008). Task instructions participate to behavioral relevance, i.e., to top-down factors influencing gaze exploration (Treue, 2003), even if it has been proposed that social information takes priority irrespective of task demands (Flechsenhar and Gamer, 2017).

While emotional recognition performance (Aguillon-Hernandez et al., 2022) and ocular exploration (this study) are affected by facial accessories, their effect is minor on physiological arousal reflected by pupil dilation. We observed a very large and robust effect of the emotional content. The emotions we studied (sadness, happiness, and neutrality) influenced the peak pupil dilation, with a greater dilation for sadness. This result is in agreement with the consensus that pupil diameter increases when emotional stimuli are observed (Bradley et al., 2008), reflecting a physiological arousal probably related to greater empathic engagement (Frith, 2009). For example, Bradley et al. (2008) showed an increase in pupil diameter when adult participants observed happy or sad images compared to neutral images (not specifically faces). More recently, Aguillon-Hernandez et al. (2020) showed that the pupils of neurotypical children were sensitive to the emotional content of the face, and especially sadness, only when the stimuli were dynamic, as in the present study. Finally, Partala et al. (2000) reported an increase in pupillary dilation when listening to emotionally valenced sounds, compared to neutral sounds, showing the influence of emotion on pupil even when the stimulus is not visual. We also observed an interaction between accessory and emotion on the peak pupil dilation, with a greater pupil dilation for sadness mainly present in the scarf and sunglasses conditions. Sadness did not seem to evoke a larger dilation than happiness when observing a masked face (even if not significantly different from sadness in the other accessory conditions). This result could be linked to the decreased performance in sadness recognition in presence of the mask (Aguillon-Hernandez et al., 2022), or to a psychological effect of the mask (Marler and Ditton, 2021; Saunders et al., 2021) that could hinder the processing of the emotional content of the face. The differences of pupil dilation in response to sadness might also reflect a difference in exposure between the accessories, with mask having become usual in our everyday life. A combination of unfamiliarity and negative emotion could possibly evoke a larger activation of the amygdala (Straube et al., 2011; Mattavelli et al., 2014), a probable source of pupil dilation modulation (see below). Further studies should explore frequency of exposure, and its link to explicit emotional recognition, to go further in the interpretation of these results.

The presence of an accessory only produced small effects on pupillary parameters, which need to be confirmed with a larger population, with a latency of the peak pupil dilation slightly longer in the mask than in the scarf condition. This latency was not correlated with the amplitude of peak dilation itself. Such a small latency effect is difficult to interpret considering the slow dynamics and integrative nature of pupil dilation. As there is no low-level difference between the mask and scarf conditions, this effect could possibly reflect a delay in the processing of masked faces due to a cognitive bias (Marler and Ditton, 2021; Saunders et al., 2021).

The robust main effect of emotion on pupil dilation, regardless of the presence and nature of the accessory, and with short video presentations of 2 s, suggests that, even if emotion recognition is hindered (Aguillon-Hernandez et al., 2022), an implicit emotional processing is still preserved. While facial emotion processing involve both cortical and subcortical regions (e.g., Williams et al., 2006), subliminal presentation or unseen stimuli would mainly activate the subcortical regions (Morris et al., 1999; Williams et al., 2006). This implicit processing would involve a fast subcortical loop (Johnson, 2005), including amygdala, participating in face detection and modulated by emotional processing. This subcortical loop could directly modulate pupil diameter via projections from the amygdala onto the reticular formation, probably in the same way subliminal fear can induce skin conductance responses (Williams et al., 2006). We propose that this fast subcortical loop implicitly processes emotional cues present on the face even when an accessory is worn, but that this emotional signal would not be sufficient for a completely preserved explicit recognition. Explicit recognition, relying on a large cortical and subcortical network (e.g., Vuilleumier and Pourtois, 2007), involves visual processing in early visual areas, thereby affected by the loss of visual input and the modification of ocular exploration, but also cognitive processing in frontal regions that could be modified by cognitive bias (Marler and Ditton, 2021; Saunders et al., 2021). This latter factor could explain the difference in emotion recognition between the scarf and mask conditions (Aguillon-Hernandez et al., 2022). The preserved automatic processing of emotion in our study is observed in expert adults, but may not be observed in children, who are not yet face experts (Diamond and Carey, 1986).

In conclusion, this study is the first to evaluate the effect of facial accessories, and in particular the COVID-19 mask, on the visual exploration and physiological reactivity to ecological emotional faces. We have shown that the COVID-19 mask alters the ocular reading of the face, but with few specific effects compared to another accessory covering the lower part of the face. The physiological adjustment to observing a masked face is slightly disturbed, with a diminished and delayed pupil reactivity. The COVID-19 pandemic brought several concerns, and in particular diminished social interaction quantitatively and qualitatively. Our ocular and pupillary results on masked faces observation point toward only a slight deleterious effect of the mask, even if emotion recognition is affected (Aguillon-Hernandez et al., 2022). Beyond the COVID-19 pandemic, studying the influence of the mask is also primordial to better understand the doctor-patient relationship. Our results in adult participants suggest that even masked, a person can convey an emotional signal perceived implicitly by the observer.

## Data availability statement

The raw data supporting the conclusions of this article will be made available by the authors, without undue reservation.

## Ethics statement

The studies involving human participants were reviewed and approved by CPP, protocol PROSCEA 2017-A00756-47. The patients/participants provided their written informed consent to participate in this study. Written informed consent was obtained from the individual(s) for the publication of any potentially identifiable images or data included in this article.

## Author contributions

CW, NA-H, and ML contributed to conception and design of the study. VR and CR worked on technical settings and subject recruitment. VR acquired and processed the data and wrote the first draft of the manuscript. VR, NA-H, and CW performed the statistical analysis. CW and NA-H wrote sections of the manuscript. All authors contributed to manuscript revision, read, and approved the submitted version.
